# Altered Ventilation in Rats With Established Severe Monocrotaline‐Induced Pulmonary Hypertension: The Role of the Dorsal Hypothalamus

**DOI:** 10.1002/cph4.70055

**Published:** 2025-09-24

**Authors:** Aliaume Le Pape, Christophe Guignabert, Ly Tu, Nathalie Mougenot, Emma Burban, Denis David, Hugo Bottemanne, Emmanuelle Corruble, Thomas Similowski, Caroline Sevoz‐Couche

**Affiliations:** ^1^ INSERM UMRS1158 Neurophysiologie Respiratoire expérimentale et Clinique, Sorbonne Université Paris France; ^2^ INSERM UMR_S 999 Hypertension Pulmonaire: Physiopathologie et Innovation Thérapeutique (HPPIT) Le Kremlin‐Bicêtre France; ^3^ Unité Mixte de Recherche en Santé (UMR_S) 999 Hypertension Pulmonaire: Physiopathologie et Innovation Thérapeutique (HPPIT), Université Paris‐Saclay Le Kremlin‐Bicêtre France; ^4^ UMS28, INSERM, Sorbonne Université Plateforme PECMV Paris France; ^5^ MOODS Team, INSERM 1018, CESP (Centre de Recherche en Epidémiologie et Santé des Populations) Faculté de Pharmacie, Bâtiment Henri MOISSAN, Université Paris‐Saclay Orsay France; ^6^ Service Hospitalo‐Universitaire de Psychiatrie de Bicêtre, Assistance Publique‐Hôpitaux de Paris, Hôpitaux Universitaires Paris‐Saclay, Hôpital de Bicêtre Le Kremlin Bicêtre France; ^7^ MOODS Team, INSERM 1018, CESP (Centre de Recherche en Epidémiologie et Santé des Populations) Faculté de Médecine Paris‐Saclay, Université Paris‐Saclay Le Kremlin Bicêtre France; ^8^ Service Des Explorations Fonctionnelles de la Respiration, de L‘exercice et de la Dyspnée du Département R3S AP‐HP, Groupe Hospitalier Pitié‐Salpêtrière Charles Foix Paris France

**Keywords:** hypothalamus, pulmonary hypertension, severity, stress, ventilation

## Abstract

Pulmonary hypertension (PH) is a progressive condition characterized by muscularization of precapillary arteries, chronic inflammation, and a loss of the distal pulmonary circulation. These changes can be mimicked in rats by monocrotaline (MCT) administration. The impact of MCT‐induced pulmonary damage on ventilatory control is poorly understood. In this study, we investigated whether the severity of PH induced by MCT in rats is linked to ventilatory changes. We examined ventilatory variables in awake (plethysmographic) and anesthetized (tracheal) rats, stratified by disease severity (Fulton's index). Two subgroups were identified: mild (Fulton's index < 0.49) and severe (Fulton's index > 0.53) PH. Resting ventilation was significantly increased in severe but not mild PH rats compared to controls, in both awake and anesthetized states. However, the responses to a carotid chemoreflex were similar between all groups. The variability of ventilatory variables was elevated in both groups of PH rats compared to controls, with a more pronounced increase in animals severely affected. Disease severity was also associated with increased adrenal gland weight, reduced brain‐derived neurotrophic factor (BDNF) levels, and prolonged grooming, suggesting a heightened arousal state in severe PH rats. Interestingly, these alterations were reversed by inhibition of the dorsomedial hypothalamus area, a key structure in stress defense responses, and the blockade of the parabrachial complex, which projects onto the central ventral respiratory group. These findings suggest that PH‐induced changes, whether due to vascular remodeling and/or right ventricular dysfunction, are associated with severity‐dependent ventilatory alterations, possibly mediated via stress‐related neural activation.

## Graphical abstract

In PH patients, exertional dyspnea may participate in stressful conditions, particularly in more advanced stages. High stress induces the activation of a DMH/PeF‐KF/PB‐CVRG neurocircuitry, at the origin of a hyperventilation which may reinforce dyspnea and stress.

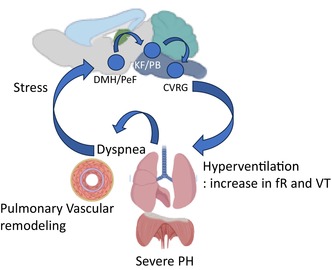

## 1 Introduction

Pulmonary hypertension (PH) is a progressive and life‐threatening condition characterized by increased pulmonary vascular resistance due to structural remodeling of the small pulmonary arteries (Tobal et al. 2021; Guignabert and Dorfmuller 2013). These changes include muscularization of precapillary arterioles, chronic perivascular inflammation, and a progressive loss of the distal pulmonary microcirculation, features that are partially mimicked in the monocrotaline (MCT) rat model, a widely used tool for studying PH pathology (Dorfmüller et al. 2011). However, the impact of these alterations on respiratory control remains poorly understood.

Beyond vascular remodeling, PH patients also exhibit hyperventilation during exercise, characterized by an excessive increase in ventilation relative to carbon dioxide production (Caravita et al. 2017). At rest, patients with pulmonary embolism are often hypocapnic, with increases in respiratory rate and tidal volume (Mélot and Naeije 2011). The presence of baseline ventilatory abnormalities—in PH remains to be confirmed. If present, it may result from a potential increase in chemoreflex peripheral sensitivity, as suggested in idiopathic hyperventilation (Jack et al. 2003). In a recent study, investigators found that resting breath‐by‐breath variability was higher in PH patients than in healthy controls (Plunkett et al. 2024). However, this breathing pattern was not associated with hemodynamic alterations or exercise capacity, so the mechanisms remain unknown. It is known that chronic stress increases breathing frequency and its variability (Kinkead et al. 2009). Patients with PH experience multiple symptoms such as dyspnea, fatigue, and sleep disturbances, which impair their health‐related quality of life (Ruopp and Cockrill 2022). Therefore, PH may induce a stress phenotype (Von Visger et al. 2018) that could, at least in part, explain the increase in some respiratory variables and their variability. A better understanding of these mechanisms is essential because they may be responsible for the persistence/amplification of dyspnea at rest and during exercise despite the optimization of treatments, which is highly debilitating and a source of supplementary stress in PH patients.

In this study, we aimed to better understand how PH influences respiratory control and whether these alterations reflect disease progression. Our first goal was to investigate whether increased ventilation minute and/or increased in respiratory variability can be observed in awake and anesthetized MCT‐induced PH rats and correlate with the severity of the disease. We examined ventilatory patterns in rats with established PH, stratifying them by disease severity based on Fulton's index. This allowed us to compare respiratory variables between rats with mild and severe PH and assess whether these changes are linked to the extent of vascular and right ventricular remodeling. Our second goal was to better understand how PH influences central respiratory control. We investigated in control, mild, and severe PH rats the ventilatory response to intravenous administration of cyanide potassium (KCN), carotid chemoreflex activation, as well as the modulation of baseline ventilation variables after the chemical blockade of the dorsomedial nucleus of the hypothalamus/perifornical area (DMH/PeF)—a key structure involved in stress‐induced hyperventilation, as well as that of the Kölliker‐Fuse/parabrachial (KF/PB) complex.

## 2 Materials

### 2.1 Ethics Statement

Experiments were carried out in 48 Sprague‐Dawley male rats weighing 290–310 g (6–8 weeks old). Ethical approval was obtained for this project (#2492). PH was induced in 28 rats by a single subcutaneous injection of MCT (60 mg/kg, Sigma‐Aldrich) in a volume of 0.5 mL on day 0 (D0). MCT was dissolved in 1 N HCl, and the pH was adjusted to 7.4 with 1 N NaOH, following previously established protocols (Tobal et al. 2021). Control rats (*n* = 23) received an injection of MCT vehicle.

### 2.2 General Preparation

Under light anesthesia (isofluorane 0.5%), echocardiography (D21), and hemodynamic measurements (D22) were performed to measure right ventricular systolic pressure (RVSP) and pulmonary vascular resistance. Pulmonary vascular resistance was estimated noninvasively using the Doppler‐derived index (PVR_Ech_), expressed in m/s/cm, calculated as: peak tricuspid regurgitant velocity [TRVmax, expressed in m/s]/time‐velocity integral of right ventricular outflow tract TVI [expressed in cm]× 10 + 0.16. This index provides a relative measure of pulmonary vascular resistance suitable for comparison between experimental groups, though it does not correspond to classical units such as dyn·s·cm^−5^ or Wood units (Abbas et al. 2003). The Fulton's index (RV/LV + S) was also calculated to assess cardiac hypertrophy (Guignabert et al. 2005). Respiratory variables in MCT‐treated rats were analyzed in relation to disease severity. Commonly, thresholds are defined based on the distribution of a variable, often using a value 2 standard deviations above or below the mean (Robinson et al. 1991; Kroc and Olvera Astivia 2022). In this study, we chose a discriminative threshold based on the Fulton's index, a widely used marker of PH severity in rodents, as it directly reflects the degree of right ventricular hypertrophy, a key consequence of sustained elevated RVSP.

### 2.3 Recordings of Ventilatory Variables

Ventilatory variables were recorded at D0 and D22 in awake animals using a whole‐body plethysmography chamber (Chen et al. 2025), and at D22 in anesthetized rats (1% isofluorane) via a tracheal cannula connected to a pneumotachograph (Fleisch 0000) coupled with a volume transducer (Gould Z46170). Resting minute ventilation (*V* *_E_*, mL/min/100 g) was calculated as: *V* *_E_* = *V* *_T_* × fR, where *V* *_T_* is the tidal volume (mL, normalized to body weight) and fR is the breathing frequency (breaths per minute, bpm). The coefficient of variation (CV) for fR, *V* *_T_*, and *V* *_E_* was calculated as standard deviation (SD)/mean × 100.

In anesthetized rats, animals were secured in a stereotaxic frame with the head in a flat‐skull position. Femoral vein and artery cannulations were performed. Anesthesia depth was monitored by paw pinch and arterial blood pressure stability. Rectal temperature was maintained at 37°C using a thermostatically controlled heating blanket. A chemoreflex challenge was performed by intravenous administration of potassium cyanide (KCN, 40 μg/rat in 0.1 mL) via the femoral vein. The chemoreflex ventilatory response was quantified as the difference between maximal values during KCN administration and baseline values. After KCN injection (30 min), microinjections of either saline or muscimol (5 mM, 0.1 μL), a selective GABA_A_ receptor agonist, were delivered into either the DMH/PeF region (P 3.0–3.5, L 0.5–1, and V 7.5–8 mm from bregma) (Zafar et al. 2018; Sévoz‐Couche et al. 2013) or the KF/PB complex (P 8.5–8.9 mm, L 2.5–3.0 mm, V 7–8 mm) (Boscan et al. 2005). Maximal changes (difference between maximal and baseline values) in ventilatory variables induced by the chemical blockade of these regions were calculated.

### 2.4 Establishment of Stress State

A stress state in the animals was assessed by measuring serum brain‐derived neurotrophic factor (BDNF) levels at D0 and D22, and by determining adrenal gland (AG) weight expressed relative to total body weight (mg/100 g BW) at D22. In addition, the body weights of control and PH rats were measured at D0, D7, D14, and D21. Self‐grooming behavior, which reflects emotional reactivity and arousal in rodents, was also monitored. Grooming was scored according to a standardized sequence (bilateral paw and nose grooming, followed by unilateral face grooming, then bilateral head grooming, and finally body licking) (Langen et al. 2011).

### 2.5 Statistics

Unpaired *t*‐tests were used to compare the Fulton's index, PVR, and RVSP between control and MCT‐treated animals. One‐way ANOVA was performed in the control, MCT low, and MCT high groups to compare echocardiographic and hemodynamic values, morphological parameters, stress parameters, and behavior, ventilation at rest or during the chemoreflex challenge in anesthetized rats. Two‐way repeated (time) measures ANOVA was used to compare ventilation and self‐grooming in awake male rats undergoing plethysmography at D0 and D22, body weight at D0, D7, D14, and D21, and serum BDNF at D0 and D21. Two‐way ANOVA (vehicle vs. muscimol) was used to compare the effect of treatment on ventilatory variables in anesthetized animals at rest.

## 3 Results

**FIGURE 1:**
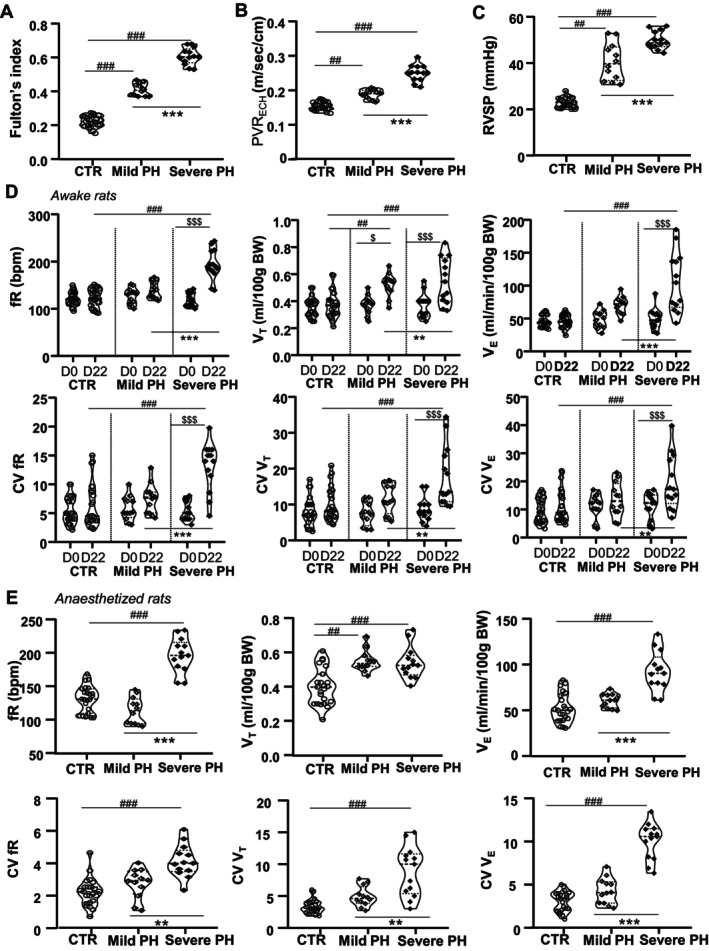
Baseline ventilation and ventilation variability in awake and anesthetized control and PH rats. Violin plots showing that severe PH rats had a higher Fulton's index (A), PVR_ECH_ (B), and RVSP (C) than mild PH and control (CTR) animals. (D, E) The ventilation minute and its variability recorded at D22 by plethysmography (D) or during anesthesia (E) are increased in Severe PH rats compared to Mild PH and CTR animals. CV fR, coefficient of variation of the respiratory frequency; CV *V* *_E_*, coefficient of variation of the minute ventilation; CV *V* *_T_*, coefficient of variation of the tidal volume; fR, respiratory frequency; PVR_ECH_, pulmonary vascular resistance from doppler echocardiography; RVSP, right ventricular systolic pressure; *V* *_E_*, minute ventilation; *V* *_T_*, tidal volume. ^##^ *p* < 0.01 and ^###^ *p* < 0.001 versus CTR; **p < 0.01 and ***p < 0.001 versus mild PH; ^$^ *p* < 0.05 and ^$$^ *p* < 0.01 versus D0.

In MCT‐injected rats, Fulton's index was significantly higher (0.51 ± 0.019, *n* = 28) than in controls (0.22 ± 0.006, *n* = 23) (Figure S1A). Using the discriminative threshold described in Methods, 3 MCT rats with a Fulton's index between 0.48 and 0.53 were excluded. Rats with a high Fulton's index (> 0.53; *n* = 13, 0.60 ± 0.01) were classified as severe PH, while those with a low Fulton's index (< 0.48; *n* = 12, 0.41 ± 0.01) were classified as mild PH (Figure 1A). Echocardiographic (Figure S1B) and hemodynamic recordings revealed higher TVI, PVR_ECH_, AT/ET, and RVSP in MCT than control animals (Figure S1C–F). TVI (Figure S1C), PVR_ECH_ (Figure 1B), and RVSP (Figure 1C), but not AT/ET (Figure S1E), were higher in severe PH than in mild PH or control rats.

**FIGURE 2:**
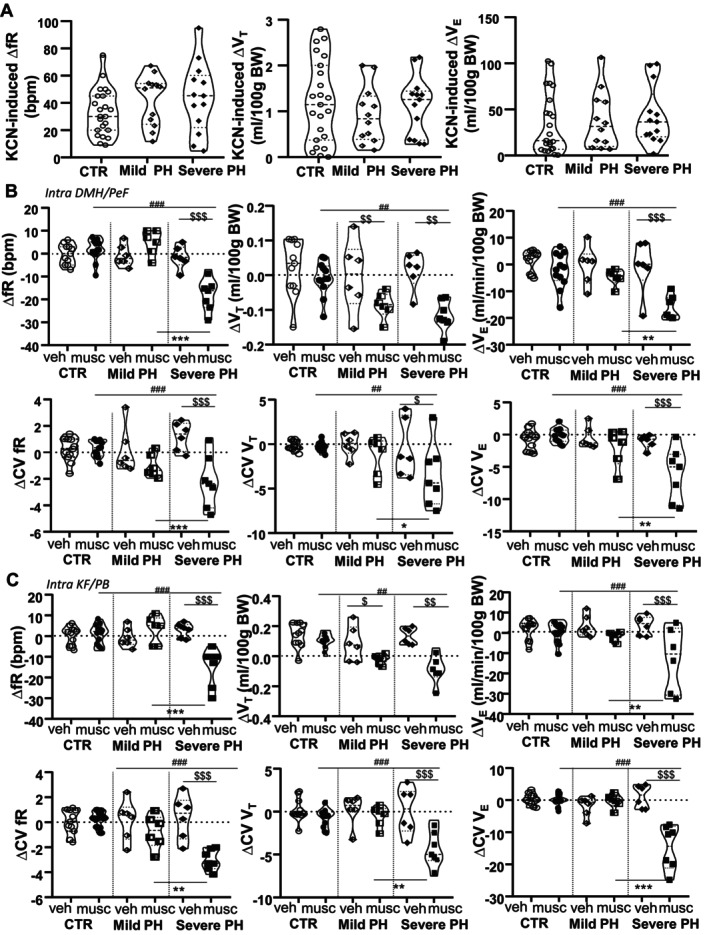
Ventilatory response at D22 during a chemoreflex challenge or after blockade of the DMH/PeF area in anesthetized rats. (A) The increase in ventilation minute induced by KCN was similar between CTR, mild and severe PH groups. (B, C) Microinjections of muscimol to block the DMH/PeF (B) or the kölliker fuse/parabrachial complex (KF/PB, C) area reduced the minute ventilation (Δ*V* *_E_*) and its coefficient of variation in severe PH rats only. CV fR, coefficient of variation of the respiratory frequency; CV *V* *_E_*, coefficient of variation of the minute ventilation; CV *V* *_T_*, coefficient of variation of the tidal volume; fR, respiratory frequency; *V* *_E_*, minute ventilation; *V* *_T_*, tidal volume.^##^ *p* < 0.01 and ^###^ *p* < 0.001 versus CTR; *p < 0.05, **p < 0.01 and ***p < 0.001 versus mild PH; ^$^ *p* < 0.05, ^$$^ *p* < 0.01 and ^$$$^ *p* < 0.001 versus D0.

In awake rats, minute ventilation (*V* *_E_*) and its CV at D22 were elevated in severe PH compared to mild PH and controls, driven primarily by increased fR and its variability (CV fR) (Figure 1D and Figure S2). Similar patterns were observed in anesthetized rats (Figure 1E). Notably, mild PH rats showed higher *V* *_T_* than controls, although minute ventilation was unchanged. The hyperventilatory response to KCN was comparable across all groups (Figure 2A).

**FIGURE 3:**
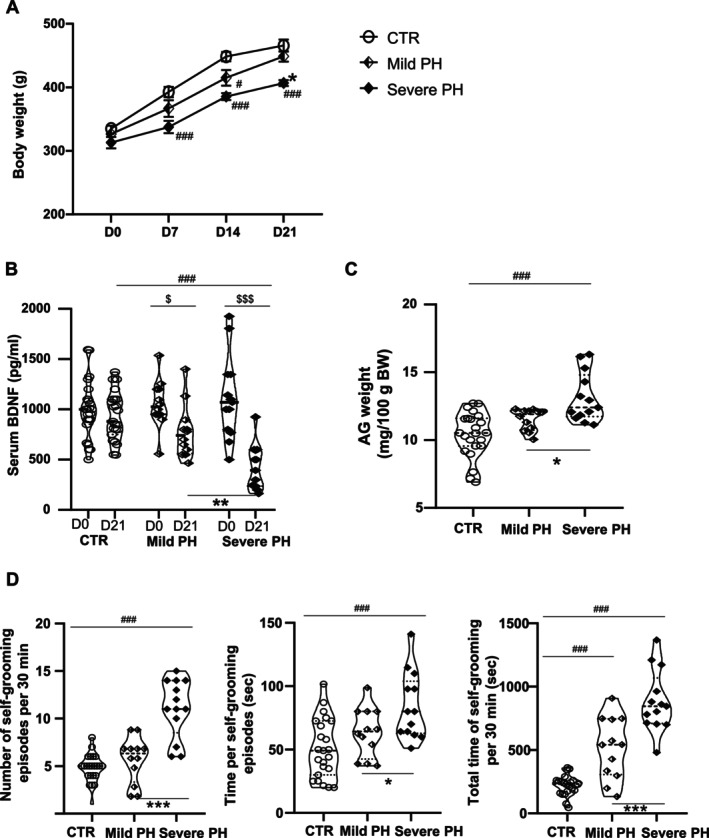
Stress markers are higher in severe than mild PH rats. At D21, the body weight gain (A) and serum BDNF levels (B) are reduced in severe compared to mild PH and CTR rats. At D22, adrenal gland (AG) weight (C) and self‐grooming (D) are higher in severe PH than mild PH and CTR animals. ^###^ *p* < 0.001 versus CTR; *p < 0.05, **p < 0.01 and ***p < 0.001 versus mild PH; ^$^ *p* < 0.05 and ^$$$^ *p* < 0.001 versus D0.

To investigate the potential involvement of central neurovegetative circuits in these ventilatory alterations, we examined physiological and behavioral markers associated with hypothalamic activation and stress‐related responses. At D22, microinjections of muscimol, but not vehicle, into the DMH/PeF area (Figure S3A) or the KF/PB complex significantly reduced *V* *_E_* and its variability in severe PH rats, primarily through decreases in fR and CV fR (Figure 2B,C) (*p* < 0.001 for both, two‐way ANOVA); representative data for the effect of DMH/PeF blockade are shown in Figure S3B. These findings were consistent with other stress indicators: decreased weight gain (Figure 3A), reduced BDNF serum levels (Figure 3B), and increased AG gland weight (Figure 3C) and self‐grooming behavior (Figure 3D). Interestingly, blockade of these regions also reduced V_T_ in mild PH, accompanied by lower BDNF levels, although these changes did not reach statistical significance compared to controls.

## 4 Discussion

In this study, we investigated the impact of MCT‐induced PH on ventilatory control and whether the severity of the disease influences these alterations. Our findings demonstrate that rats with severe PH exhibit marked changes in baseline ventilation and ventilatory variability compared to controls and mild PH rats. These alterations were evident under both awake and anesthetized conditions, suggesting a robust shift in respiratory regulation that is not merely a consequence of behavioral arousal, environmental stress, or anesthesia. Importantly, these ventilatory changes correlated with signs of enhanced neurovegetative drive, including increased adrenal weight, reduced circulating BDNF levels, and prolonged grooming behavior, markers commonly associated with heightened hypothalamic activation.

MCT reproduced the hemodynamic and cardiomorphometric alterations typically reported in experimental PH (Nogueira‐Ferreira et al. 2015). However, in the present study, we observed variability in disease expression, which allowed retrospective classification of rats into mild and severe PH groups based on Fulton's index. Several factors may contribute to this heterogeneity, including rat strain (Sprague–Dawley rats are less severely affected than Wistar (Mathew et al. 1989)), protocol duration (vascular lesions progress up to 4–5 weeks (Hołda et al. 2020)), and variability in MCT metabolite toxicity (MCT pyrrole), which depends on oxidative stress and inflammatory responses (Dahal et al. 2011). Finally, although these factors may partly account for the variability observed, a technical effect related to the experimental procedures cannot be completely excluded.

Patients with severe PH often experience symptoms such as dyspnea, fatigue, and sleep disturbances (Laveneziana et al. 2013), all of which contribute to impaired health‐related quality of life (Ruopp and Cockrill 2022). Our data show that severe PH rats exhibit a higher respiratory frequency combined with elevated tidal volume, resulting in increased minute ventilation, a pattern consistent with hyperventilation. However, only a direct measurement of PaCO_2_ would confirm whether hypocapnia, and thus true hyperventilation, was present. The relationship between baseline ventilatory changes and disease severity indicates that respiratory control is not passively altered by PH‐related hypoxia or mechanical loading but is actively modulated in a manner that scales with the extent of vascular remodeling. We first explored the possibility that an increased sensitivity to chemoreflex challenge in severe PH rats could explain these changes (Plunkett et al. 2024), but KCN hyperventilatory responses were similar in all groups of rats. Another possibility was that a higher state of stress in rats with severe PH could be at the origin of the ventilatory changes. Psychological conditions such as depression, anxiety, and panic disorder are more common in PH patients than in the general population (Von Visger et al. 2018). Patients with more severe PH may experience higher levels of psychological stress and a lower quality of life (Zhang et al. 2024). Respiratory rate is known to be higher in high‐anxiety compared to low‐anxiety behavior male rats (Carnevali et al. 2013). Kinkead and collaborators showed that male rats subjected to neonatal maternal separation had higher frequency and tidal volume respiratory instability (Kinkead et al. 2009, 2013). In agreement, our results demonstrate that MCT increases physiological stress markers, with a stronger effect in severe PH: reduced body weight gain, decreased serum BDNF (Ihara et al. 2016; Brouillard et al. 2020; Blugeot et al. 2011), enlarged AGs (Sévoz‐Couche et al. 2013; Tran and Gellner 2023), and enhanced grooming behavior. The effect of stress on self‐grooming can often be described as an inverted U‐shaped function: self‐grooming typically becomes longer during moderate arousal (as a ‘displacement activity’) and can be inhibited by high‐stress states that elicit freezing, fight or flight responses (Kalueff et al. 2016). Here, the number of self‐grooming episodes and the time per episode were higher in rats with severe PH compared to mild and control groups. To note, in mild PH, BDNF levels were decreased at D21 compared to D0, and the total time of self‐grooming episodes was higher than in controls. Taken together, these data suggest that animals with severe PH are significantly affected by stress, whereas those with mild PH exhibit only minor stress symptoms.

These findings suggest that stress‐induced CNS circuits contribute to PH‐related ventilatory alterations. A key component is the hypothalamo‐pituitary–adrenal (HPA) axis, which orchestrates adaptive responses to stress. The HPA axis is integral to the regulation of the defense response and returning the body to homeostasis after exposure to a traumatic stressor (Herman et al. 2016). A subcortical defense system is well adapted to responding to threats that require an immediate stereotyped response that does not involve the cortex. The basal ganglia/colliculi system is phylogenetically ancient. In contrast, the defense system that includes the DMH/PeF area—a region implicated in sympathetic activation, behavioral responses, and cardiorespiratory integration in different models of chronic stress (Sévoz‐Couche et al. 2013; Brouillard et al. 2020; DiMicco et al. 2002; Bondarenko et al. 2015)—and cortex evolved at a later time and appears to be better adapted to generating appropriate responses to more sustained threatening stimuli (Dampney 2015). The activation of the DMH/PeF exerts a powerful stimulatory effect on respiratory activity (increase in frequency and amplitude of the phrenic nerve activity), causing hyperventilation (McDowall et al. 2007). Here, we show that targeted inhibition of the DMH/PeF with muscimol can reverse the main respiratory alterations observed in rats with severe PH and normalized ventilatory variability. In mild PH, blockade of this region reduced *V* *_T_*, suggesting that this tidal volume reduction may be associated with stress. These results provide direct evidence that the DMH/PeF area plays a pivotal role in mediating the central effects of severe PH on respiratory control. Increased sympathetic nervous system activity, known to occur in PH (Liu et al. 2017), is associated with central injury in other conditions (Wei et al. 2018; Taylor et al. 2018). In particular, the hippocampus, which plays a role in shutting off the HPA stress response (Roy et al. 2021), is injured and may lead to a sustained DMH activation (McEwen 2007). However, the DMH/PeF area doesn't project directly to the central ventral respiratory group (CRVG, responsible for respiratory drive), so an intermediate region may be involved in the DMH/PeF‐induced respiratory alteration. The KF/PB complex was a good candidate because it received projections from the DMH and its efferents reach the CRVG (Chamberlin and Saper 1994; Moga et al. 1990). In addition, this complex is involved in the resting ventilation, with an impact on both fR and *V* *_T_* (Damasceno et al. 2015). Therefore, we examined the implication of the KF/PB complex in the hyperventilatory responses in severe PH rats. Similarly to the DMH/PeF area, inhibition of the KF/PB complex normalized ventilation in severe PH rats only.

In conclusion, our results show that MCT in rats causes a breathing pattern alteration associated with stress, including increased and instable minute ventilation. These changes correlate with disease severity and the associated stress state. In severe PH, compared to mild animals, greater vascular and cardiac remodeling may lead to more pronounced exertional dyspnea, which could extend to rest, potentially exacerbating stress and higher resting ventilation, and negatively impacting patients' quality of life. Although these findings suggest a potential role for hypothalamic/parabrachial circuits in the ventilation pathophysiology of PH, they are based on a single preclinical model. Future studies should investigate whether modulating DMH/PeF activity could offer new therapeutic avenues. Furthermore, the observation that women are more likely to develop PH, yet generally have better survival outcomes than men—a phenomenon known as the “sex paradox” (DesJardin et al. 2024)—highlights the importance of investigating sex‐specific physiological responses. Moreover, the respiratory system is also influenced by sex hormones, such as progesterone, and females show distinct ventilatory patterns in response to stress (Kinkead et al. 2009, 2013; Gargaglioni et al. 2019). Future studies should specifically address these sex‐related differences, which could refine our understanding of ventilatory adaptations in PH.

## Conflicts of Interest

The authors declare no conflicts of interest.

## Supporting information


**Figure S1:** Assessment of right ventricular hypertrophy and change in pulmonary echographic and hemodynamic in PH rats.
(A) Fulton's index was higher in PH animals with MCT than in controls.
(B) Representative echocardiographic Doppler recordings from the pulmonary artery outflow tract collected from rats at 21 days after MCT injection. Shapes and flow pattern of right ventricle outflow were altered in mild and severe PH animals, with the apparition of a mid‐systolic notch (right arrow). Blue Line: ejection time; Yellow line: acceleration time.
From echocardiography, we observed that TVI was higher in MCT rats, with severe PH statistically different from mild PH animals (C). Mild PH rats have higher TVI than controls. PVR_ECH_ (D) and AT/ET (E) were higher in MCT rats than in controls.
(F) Hemodynamics showed that RVSP was higher in rats with PH.
Data are presented as mean ± SEM or Violin Plots.
^##^ *p* < 0.01 and ^###^ *p* < 0.001 versus CTR; ***p < 0.001 versus mild PH.
AT/ET, acceleration time to ejection time ratio; PVR_ECH_, pulmonary vascular resistance obtained from doppler echocardiography; RVSP, right ventricular systolic pressure; TVI, time‐velocity integral.

**Figure S2:** Representative raw plethysmography recordings.
Raw whole‐body plethysmography traces from experimental groups showing breathing frequency (fR) and tidal volume (*V* *_T_*) differences in controls, mild, and severe PH.

**Figure S3:** The effect of DMH/PeF chemical blockade on tracheal ventilation.
(A) Representative histological sections showing the localization of microinjection sites within the dorsomedial hypothalamus/perifornical area (DMH/PeF). Injection tracks were visualized and verified relative to anatomical landmarks to confirm accurate targeting.
(B) Raw data showing the decrease in fR (mild and severe PH groups) and *V* *_T_* (severe PH rats only) obtained by tracheal recordings under anesthesia, after muscimol administration into the DMH/PeF area.

## Data Availability

The data that support the findings of this study are available from the corresponding author upon reasonable request.
