# Supplementary figures and images for: Altered Ventilation in Rats With Established Severe Monocrotaline‐Induced Pulmonary Hypertension: The Role of the Dorsal Hypothalamus
Source: Compr Physiol. 2025 Sep 24;15(5):e70055. doi: 10.1002/cph4.70055 (PMC12459422; doi:10.1002/cph4.70055)

A

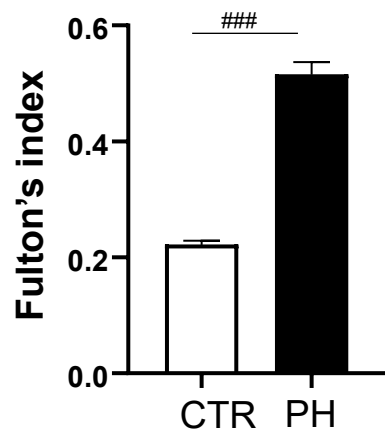

B

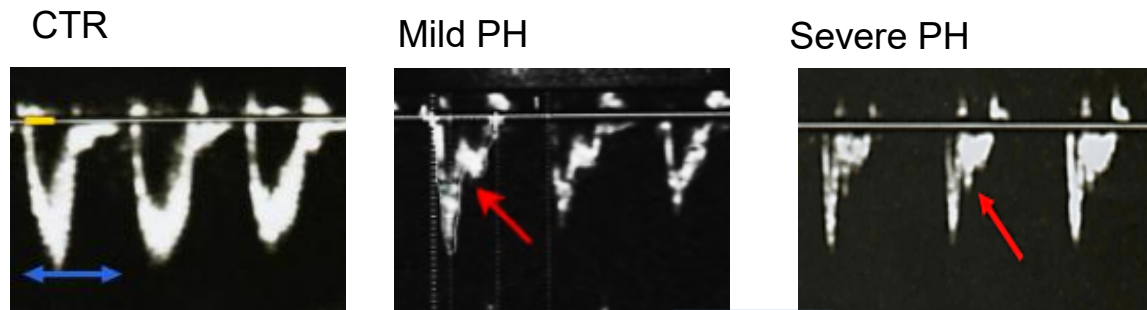

C

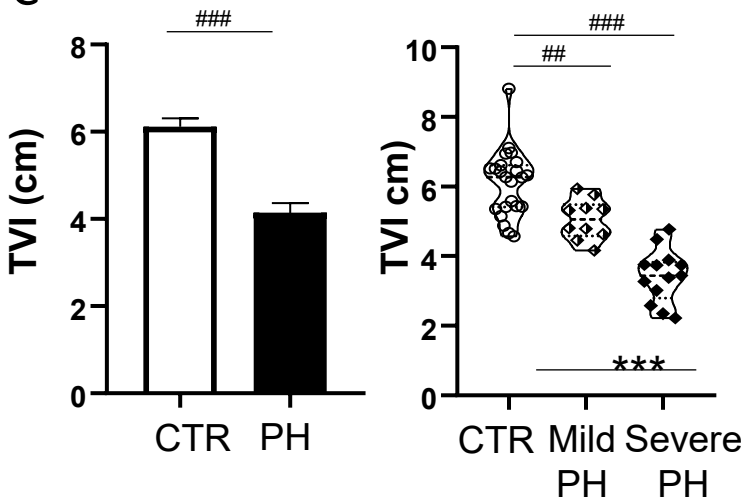

D

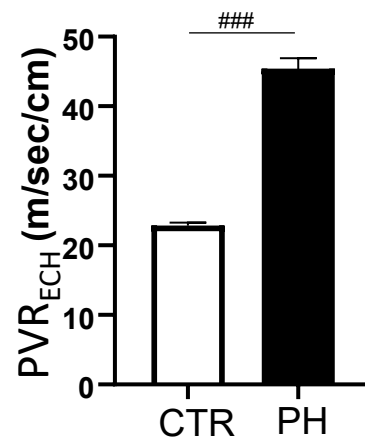

E

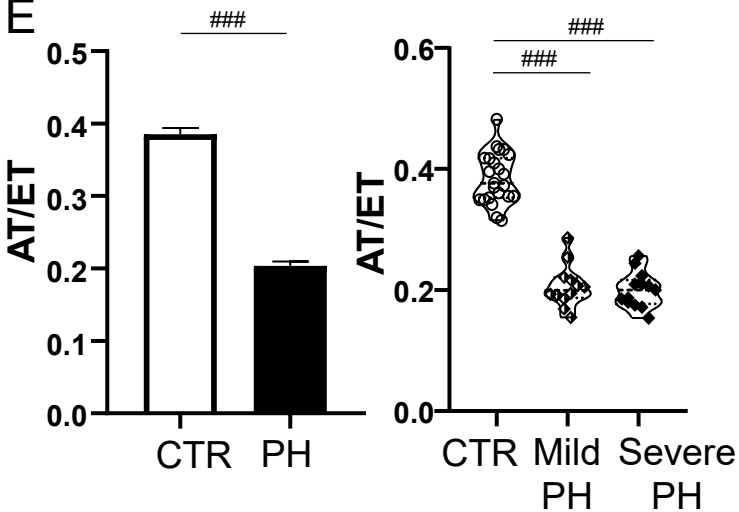

F

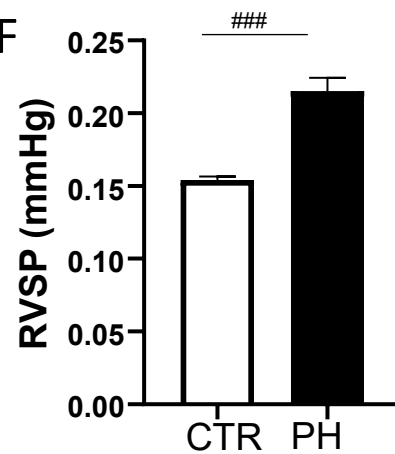

Supplement: Supplementary file 1 — Figure S1: Assessment of right ventricular hypertrophy and change in pulmonary echographic and hemodynamic in PH rats. (A) Fulton's index was higher in PH animals with MCT than in controls. (B) Representative echocardiographic Doppler recordings from the pulmonary artery outflow tract collected from rats at 21 days after MCT injection. Shapes and flow pattern of right ventricle outflow were altered in mild and severe PH animals, with the apparition of a mid‐systolic notch (right arrow). Blue Line: ejection time; Yellow line: acceleration time. From echocardiography, we observed that TVI was higher in MCT rats, with severe PH statistically different from mild PH animals (C). Mild PH rats have higher TVI than controls. PVRECH (D) and AT/ET (E) were higher in MCT rats than in controls. (F) Hemodynamics showed that RVSP was higher in rats with PH. Data are presented as mean ± SEM or Violin Plots. ## p < 0.01 and ### p < 0.001 versus CTR; ***p < 0.001 versus mild PH. AT/ET, acceleration time to ejection time ratio; PVRECH, pulmonary vascular resistance obtained from doppler echocardiography; RVSP, right ventricular systolic pressure; TVI, time‐velocity integral. [file CPH4-15-e70055-s003.pdf]

**Plethysmographic recordings at D22****Control rat**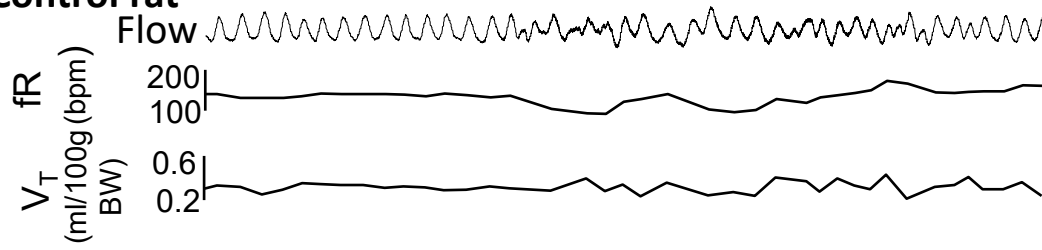**Mild PH rat**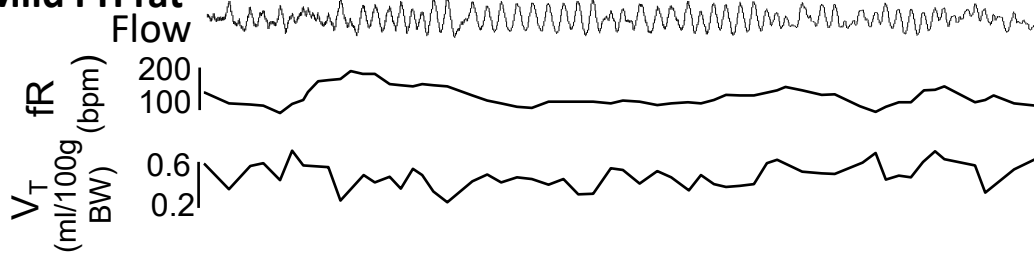**Severe PH rat**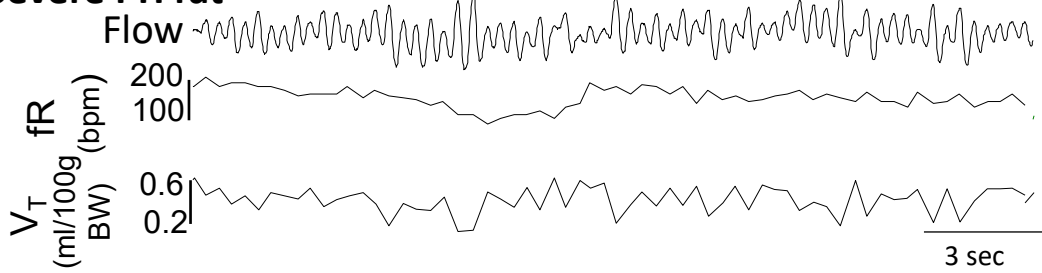

Supplement: Supplementary file 2 — Figure S2: Representative raw plethysmography recordings. Raw whole‐body plethysmography traces from experimental groups showing breathing frequency (fR) and tidal volume (V T ) differences in controls, mild, and severe PH. [file CPH4-15-e70055-s001.pdf]

A

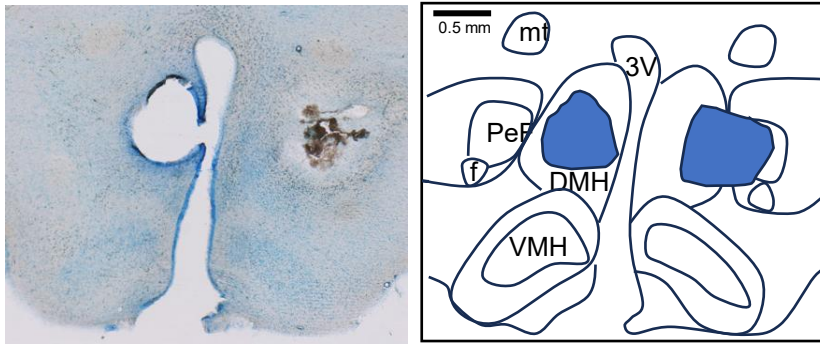

B

### Tracheal recordings at D22

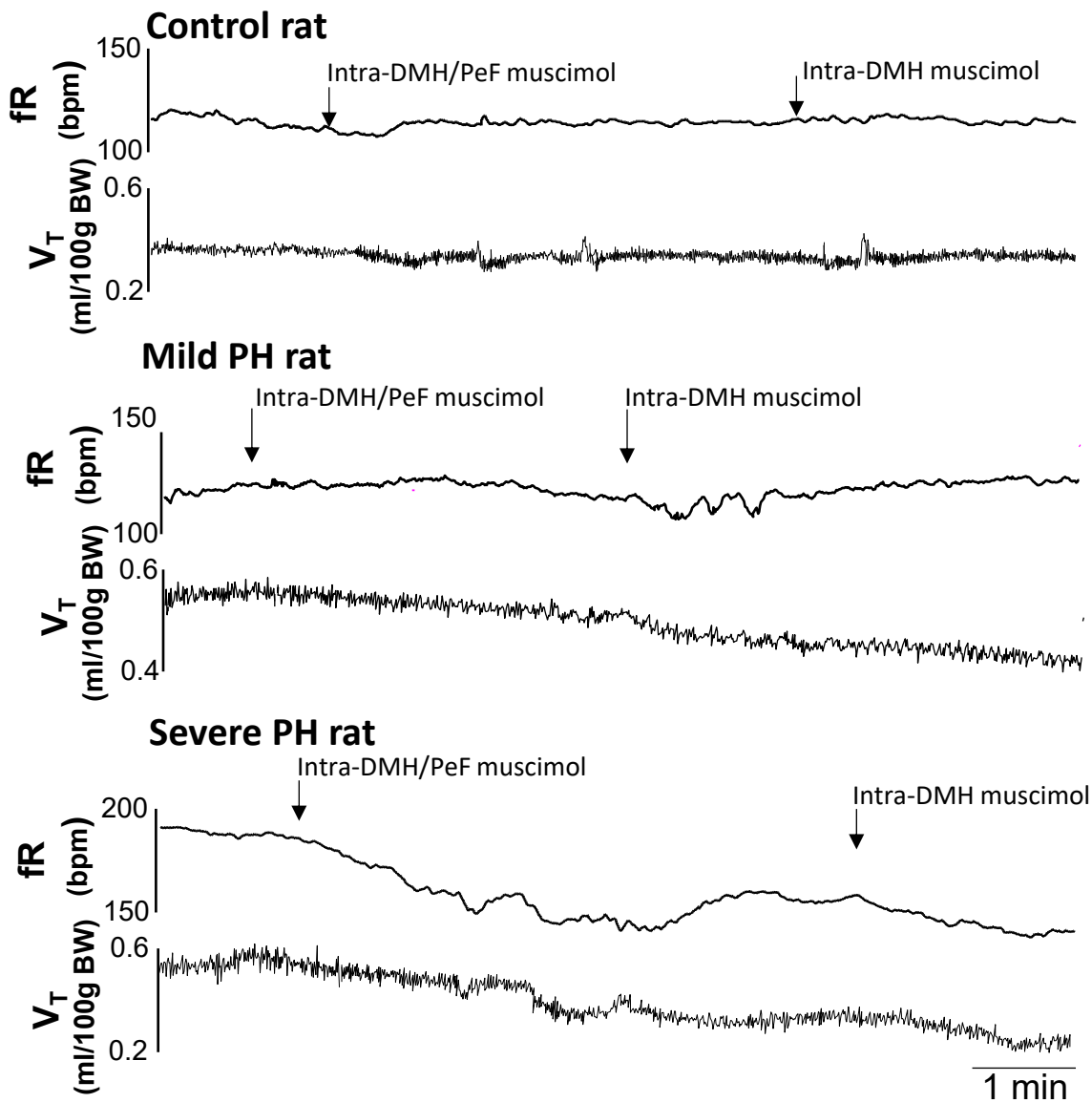

Supplement: Supplementary file 3 — Figure S3: The effect of DMH/PeF chemical blockade on tracheal ventilation. (A) Representative histological sections showing the localization of microinjection sites within the dorsomedial hypothalamus/perifornical area (DMH/PeF). Injection tracks were visualized and verified relative to anatomical landmarks to confirm accurate targeting. (B) Raw data showing the decrease in fR (mild and severe PH groups) and V T (severe PH rats only) obtained by tracheal recordings under anesthesia, after muscimol administration into the DMH/PeF area. [file CPH4-15-e70055-s002.pdf]
